# Gap Junction Channels of Innexins and Connexins: Relations and Computational Perspectives

**DOI:** 10.3390/ijms20102476

**Published:** 2019-05-19

**Authors:** Alejandro Sánchez, Carlos Castro, Dora-Luz Flores, Everardo Gutiérrez, Pierre Baldi

**Affiliations:** 1 Facultad de Ciencias, Universidad Autónoma de Baja California, Ensenada, Baja California 22860, Mexico; alejandrosg@uabc.edu.mx (A.S.); everardo.gutierrez@uabc.edu.mx (E.G.); 2 Facultad of Ingeniería, Arquitectura y Diseño, Universidad Autónoma de Baja California, Ensenada, Baja California 22860, Mexico; carlos.castro98@uabc.edu.mx; 3 Department of Computer Science, Institute for Genomics and Bioinformatics, and Center for Machine Learning and Intelligent Systems, University of California, Irvine, CA 92697, USA

**Keywords:** connexin, innexin, gap junctions, leech, central nervous system, machine learning

## Abstract

Gap junction (GJ) channels in invertebrates have been used to understand cell-to-cell communication in vertebrates. GJs are a common form of intercellular communication channels which connect the cytoplasm of adjacent cells. Dysregulation and structural alteration of the gap junction-mediated communication have been proven to be associated with a myriad of symptoms and tissue-specific pathologies. Animal models relying on the invertebrate nervous system have exposed a relationship between GJs and the formation of electrical synapses during embryogenesis and adulthood. The modulation of GJs as a therapeutic and clinical tool may eventually provide an alternative for treating tissue formation-related diseases and cell propagation. This review concerns the similarities between *Hirudo medicinalis* innexins and human connexins from nucleotide and protein sequence level perspectives. It also sets forth evidence of computational techniques applied to the study of proteins, sequences, and molecular dynamics. Furthermore, we propose machine learning techniques as a method that could be used to study protein structure, gap junction inhibition, metabolism, and drug development.

## 1. Introduction

Gap junctions (GJs) are intercellular cytoplasmic channels composed of an arrangement of transmembrane proteins particular to metazoans. These proteins form hemichannels which, while unpaired, provide a leakage passage for cytosolic molecules (like glutamate and ATP) into the extracellular medium [1]. These channels allow the exchange of intracellular ions, second messengers, and small metabolites (1 to 2 kDa) and the passage of direct current across adjacent cells [2,3,4]. Thus, current-transferring conduits provide a fundamental path for distributing electrical synapses and coordinating cellular signaling [5,6].

**Figure 1:**
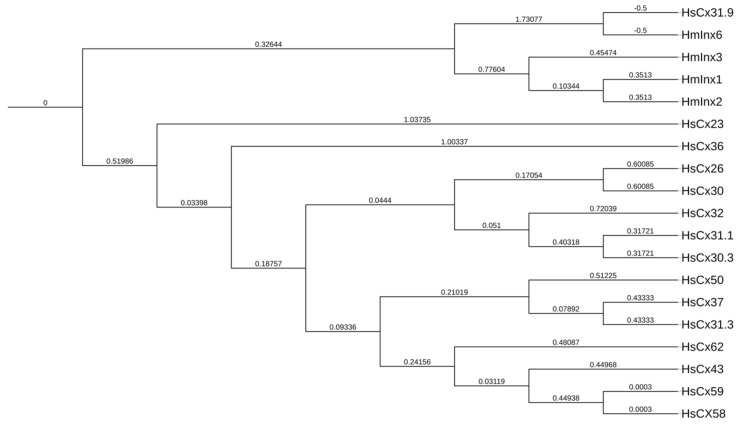
Innexin and connexin relationship dendrogram (nucleotide sequence alignment) using ClustalW. Cx31.9 presented the highest level of similarity with leech innexins. It is found on chromosome 17 and expressed in several vital organs, such as the cerebral cortex, heart, liver, and lungs. Cx31.9 presents some unique functional properties and voltage behaviors.

Currently, three main members of the gene superfamily make up these hemi-channels: connexins, pannexins, and innexins. While genetic orthology may not be evident in relating the vertebrate gap-junction connexins with their invertebrate counterpart (innexins), sequence identity analysis has identified protein homologues within the genome of several vertebrates: pannexins [2,7,8,9]. Although the junctional role of pannexins has remained questionable, their permeability to ATP, as well as their specific distribution throughout erythrocytes which do not form GJs, indicates an alternative role to that of direct intercellular communication [1,10]. However, structure-wise, the three families encode a similar topology composed of four transmembrane domains forming two extracellular loops and one intracellular loop, as well as a cytoplasmic N- and C- terminus [11,12,13]. Figure 1 is a nucleotide sequence alignment that shows the similarity between innexins and connexins using a dendrogram to categorize the relationship between those proteins. The human connexin Cx31.9 (GJA11) presented the highest level of similarity with leech innexins. Cx31.9 is expressed in several human tissues, as well as in muscle [14,15], and it exhibits very low unitary conductance and low sensitivity to transjunctional voltage [16,17]. Additionally, it has been reported that Cx31.9 plays no role in AV-nodal impulse delay or conduction elsewhere in the human heart [16].

In the case of connexins and innexins, large genetic families have been identified throughout several animal models. In mammals, out of the 20 connexin genes present in mice (*Mus musculus*), 19 can be arranged as orthologous pairs with the 21 connexins present in humans [18,19]. Meanwhile, in zebrafish (*Danio rerio*), up to 37 connexin genes have been characterized. This is the largest connexin gene family described thus far [19,20]. For invertebrates such as the fruit fly (*Drosophila melanogaster*), eight innexins encoding different loci have been identified with multiple splice isoforms [21,22]. In the nematode (*Caenorhabditis elegans*) and in the medicinal leech (*Hirudo medicinalis*), up to 25 and 21 innexins have been determined and localized, respectively [12,13,23].

In this review, we focused on the leech nervous system as a biological model to understand the human nervous system. Then, we described the morphological comparison of the molecular constituents of vertebrate and invertebrate gap junctions (connexins and innexins), as well as a description of different techniques used for inhibiting cell-to-cell communication and blocking individual channels. We propose computational methods that could be used to study protein structure, gap junction inhibition, metabolism, and drug development.

## 2. The Leech Nervous System: A Chain of Possibilities

As with most other annelids, the basic nervous system of the medicinal leech (*Hirudo* spp.) consists of a single nerve cord which runs along the ventral side of the body [24,25]. Amid both peripheral ganglia lie 21 segmental ganglia, each possessing approximately 400 neurons arranged in a tubular fashion around a central glial neuropil that provides nourishment and structure to the ganglion. Any individual neuron within the ganglion may contain the neurotransmitters acetylcholine, octopamine, gamma-aminobutyric acid (GABA), serotonin, and dopamine, as well several neuropeptides, such as met-enkephalin (mENK), FMRF-amide, bombesin, vasoactive intestinal polypeptide (VIP), and substance P [26,27,28]. The coordination of these signaling molecules alongside segmental ganglia provides the fundamental basis of hierarchical behavior patterns (feeding, swimming, and crawling) [25,29,30,31]. In addition, out of 21 innexins identified in *H. medicinalis*, 15 have been found to be exclusively expressed across the nervous system in both neurons and glial cells [13,32,33]. Moreover, during *H. medicinalis* development, specific innexins are highly expressed at certain age stage or tissue type [33].

**Figure 2:**
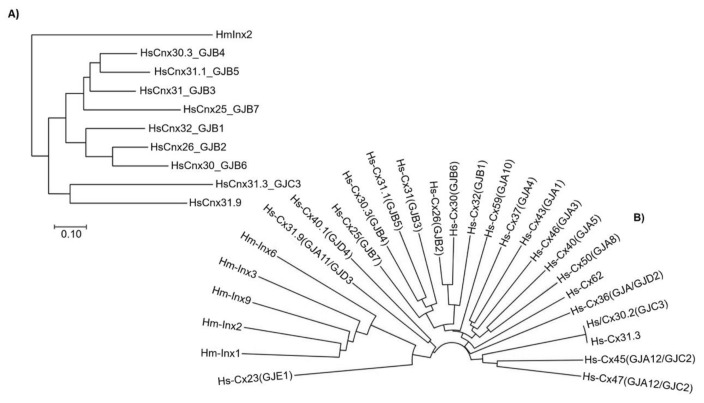
Evolutionary relationships of taxa (protein sequence alignment). Comparing (**A**) HmInx2 vs. human connexins. (**B**) HmInx1, 2, 3, 6, 9, and 14 vs. human connexins. This amino acid sequence alignment shows that Cx31.9 (GJA11) and Cx23 (GJB1) are closest to the *H. medicinalis* innexins family.

With the purpose of studying and describing GJs’ coupling patterns, as well as their relation to synaptic transmission, numerous models have been proposed and assayed. However, few have proven to be as efficient and practical as the medicinal leech nervous system, in part due to the similarity between human connexins and leech innexins [25,28]. Figure 2 shows the taxa relationships between human connexins and leech innexins. A comparison between HmInx2 and its closest human connexins is shown in Figure 2a, while a full comparison is shown in Figure 2b. As several studies have indicated, gap junction regulation may serve as a recognition mechanism that mediates the formation of electrical synapses during the embryonic development of *H. medicinalis* [12,32,34]. This suggests that electrical coupling not only precedes chemical synaptogenesis but may, in fact, lay the foundation through which transient neuronal circuits formed by interactions of complementary synaptic targets are eventually rectified through the emergence of chemical synapses during development [13,35,36]. Not restricted to synaptic coupling, this signaling mechanism has been proven to regulate and allocate glial network formation through the expression of particular innexin hemichannels [13,33,34].

For research focused around the underlying mechanisms of synaptic transmission and neurochemistry, as well as neuronal development and regeneration, the medicinal leech may be a suitable model for analyzing cell-to-cell adhesion and communication. It may also be an elegant approach to behavioral neuroscience beyond the capabilities of the discrete, yet overly simplistic, neuronal circuits of models such as *D. melanogaster* or *C. elegans* [25]. Given that numerous response mechanisms have been identified and described to be associated with a limited population of identifiable neurons whose innexin profiles have been determined within the leech, ethological research value has been placed on GJs and their neuronal wiring capabilities [25,30,37].

## 3. The Molecular Structure of Connexins/Innexins

**Figure 3:**
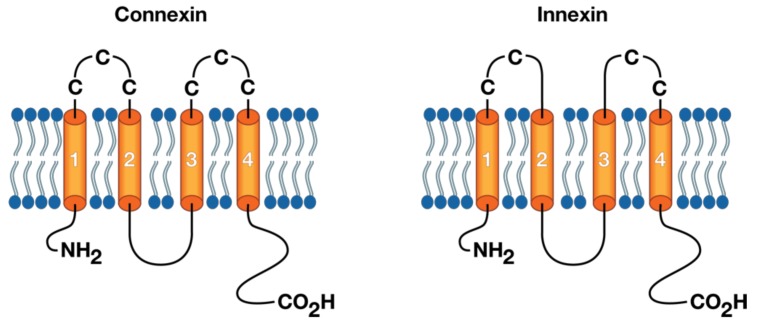
General connexin/innexin structures forming gap junctions and “hemi-channels”. Vertebrates connexins have three cysteine residues (left) in each of their extracellular loops, while invertebrates innexins have only two cysteines per loop (right). Both connexins and innexins have four transmembrane domains (orange tubes) connected by one cytoplasmic loop (black curve), and have both NH_2_ and CO_2_H terminals in the cytosol.

As previously stated, hemichannel composition consists mainly of a hexameric arrangement of connexin/innexin monomers into a cylindrical channel with a central axial pore, the coupling of which produces a functional intercellular gap junction. Though every innexin isoform varies to a certain degree in terms of sequence identity, at a structural level they appear consistent in terms of the following features: tetra spanning α-helical transmembrane segments (TM1–TM4), one cytoplasmic loop (CL), and two extracellular loops (E1 and E2) [4,8]. Figure 3 depicts a general connexin/innexin structure showing the cysteine residues in the extracellular loops. Amino- and carboxy-termini reside within the intracellular face of the junctional membrane, where they assemble into cytoplasmic domains that confer multiple gating and selectivity properties to the gap junction. Though the junctional membrane’s significance in cell recognition may not be immediately evident at the monomer level, together with the C-terminus and the cytoplasmic loop, it provides the highest degree of size variation among the monomer isoforms [37,38,39,40].

Extracellular loops, located between TM1 and TM2 and TM3 and TM4, have been shown to function as docking sites between complementary GJs through the disulfide bonding of three cysteines in each connexin loop or two in each innexin loop [40,41,42]. Structurally, both loops possess a highly conserved amino acid sequence among connexins (except Cx31), with a [C–X6–C–X3–C] pattern for E1 and [C–X5–C–X5–C] pattern for E2 [18,42]. Therefore, docking specificity is not thought to arise from sequence-specific coupling, but from a complex arrangement of antiparallel β sheets connected by disulfide bonds into concentric β barrels [42,43]. This structural hypothesis translates accordingly into innexins, where β-sheet “hairpins” accumulate around E2, while E1 creates a constriction ring around the axial pore through a small α-helix [44]. To properly understand how these molecular structures provide recognition, gating, and flexibility, a higher-order analysis must be performed from the monomer into the hexameric hemichannel or, better yet, into the dodecameric oligopeptide that is the gap junction.

## 4. The Molecular Structure of Gap Junction Proteins

Throughout the hemichannel configuration, the orientation of α-helices surrounding the axial pore aligns predominantly in a clockwise fashion, with only a couple of right-handed segments lining the pore into a crisscrossed, tilted pattern [42,45]. Several models have aspired to predict an ion permeability mechanism based on this tilting, where structural occlusion of the pore would occur through the twisting constriction of these helices in a manner similar to that of an iris diaphragm in a camera [43,45,46] However, recent X-ray analysis performed on connexin-mediated GJs revealed no structural variation between Ca^2+^-bound and Ca^2+^-free channels, suggesting the existence of a cation exclusion mechanism based on electrostatic interactions instead [47].

Once properly assembled through the alignment of bundled transmembrane segments, hemichannel functionality depends on the coordinated interaction of numerous molecular domains [44]. Within the cytosol, N-terminal regions form a funnel-like structure around the pore entrance at the transmembrane region, restricting its diameter and effectively determining permeability properties, such as molecular cutoff size and charge selectivity. It also determines channel activity properties, such as transjunctional voltage gating [44,47,48]. Amino acid residues located at the first cytoplasmic positions have revealed a sensor-like role in determining conductance and channel polarization, allowing for a highly sensitive voltage-dependent gating. This is known as fast-gating [39,48,49]. Simultaneously, a cytoplasmic dome composed of the CL and C-terminal domain creates an entrance that acts as a harness for the N-terminal loops, associating structural functionality with the pore funnel through the intercalating habit of N-terminal α-helix and TM1 [44]. The structure of gap junction channels and the differences between connexins and innexin GJs have been reviewed [50].

## 5. Inhibition of Gap Junction Communication

GJs can be inhibited at a certain level by modifying the proteins of innexins and connexins or their corresponding RNA [51]. Different methods can be used to target the proteins with chemical agents or with antibodies, and other methods target and block the mRNA to cut the translation to proteins [52,53]. The results and characteristics of some techniques are described below.

### 5.1. Chemical Mechanisms

Chemical agents have been used to block and uncouple gap junction communication (GJC) between cells. Octanol, heptanol, and arachidonic acid are efficient reagents used to block GJC in different organisms with a variety of biological purposes [51,54]. These compounds block action potentials in the membrane by increasing junctional resistance [55].

There is evidence of GJC inhibition achieved using heptanol and arachidonic acid. GJC was blocked using heptanol in Planaria [54], the sea anemone [56], and mice [57]. GJC was also blocked using arachidonic acid in leeches [58] and with both heptanol and arachidonic acid in the sea anemones [59].

When using octanol for inhibiting GJC, connexon downregulation was demonstrated in different insects, like Oncopeltus, Hyalophora, Drosophila, Xylocopa, and Periplaneta [60,61,62,63]. In addition, GJC was inhibited in rodents to better understand the function of proteins and transcriptional regulators, as well as the mechanism of function of these inhibitors on cell communication [64,65,66]. A review of gap junction blockers in animal models in relation to seizures which includes a comprehensive list of inhibitors, can be found in [51].

Chemical mechanisms excel at reversibly blocking GJC, although these blockers are non-specific for different innexins and connexins and need a precise concentration to properly inhibit GJC [65].

### 5.2. RNA Interference

Another technique that is used to inhibit GJC is RNA interference (RNAi). RNAi decreases or eliminates a target mRNA by injecting the cell with a specific double-stranded RNA (dsRNA), thus preventing the translation of mRNA into a protein [67]. Due to the protein nature of GJs, RNA interference is a suitable approach to inhibiting GJC by knocking down innexin or connexin genes [25].

Previous results conclude that connexin mimetic peptides represent the only specific inhibitors for gap junction channel function, except for small interfering RNA [68]. Nevertheless, their use has been limited due to their low efficacy of inhibition (40–50%) and slow onset of action, while a faster action has been observed in paired oocytes when peptides were applied to single oocytes before the pairing. However, the most updated literature reports some cases where the use of siRNA has a higher percentage of inhibition. For example, Cx43 expression and, thus, channel functionality have been successfully suppressed (around 70%) using RNAi-based approaches in human bone marrow stromal cells [69]. The same Cx43 was suppressed by approximately 60% in human bronchial fibroblasts [69], 90% in human pulmonary endothelial cells [70], and 90% in mouse 3T3 fibroblasts and HL-1 cardiomyocytes [71]. In all these experiments the gap junction was significantly decreased by 50%. Other cases have been reported where the inhibition of connexins using siRNA is not so high. For example, Cx37, Cx40, and Cx43 were inhibited using siRNA in human umbilical vein endothelial cells, representing 40%, 31.5%, and 32.7% of maximal inhibition, respectively [72].

In leeches, RNAi was used to decrease the expression of the innexin Hm-inx1, reducing gap junction expression in individual neurons by more than 80% [52]. In the desert locust (*Schistocerca gregaria*), a decrease of expression levels in Inx1 (74%), Inx2 (85%), Inx3 (95%), and Inx4 (65%) genes was obtained compared to the controls [73]. In *Anopheles* mosquitoes, gene knockdown of innexin AGAP001476 mRNA gene was achieved with dsRNA at a 60% level [74].

In the mosquito *Aedes aegypti*, using dsRNA resulted in a significant knockdown of Inx1 (32%), Inx2 (69%), Inx3 (51%), Inx4 (71%), and Inx7 (86%), as well as a very low percentage in Inx8 knockdown [75]. Similar to the latter study, an *Aedes aegypti* injection of Inx2 dsRNA resulted in a reduction of 73% in mRNA expression, producing a different reduction level in different tissues. There was a knockdown of Inx2 in the midgut (95%), ovaries (89%), fat body (91%), and malpighian tubules (45%) [76].

Contrary to the chemical mechanisms, RNAi has the advantage of being specific for a given innexin target due to the specificity of the dsRNA to the mRNA. Nonetheless, the reduction percentage in the expression of the genes can vary in different innexins, organisms, and even tissues.

### 5.3. Anti-Peptide Antibodies

Anti-peptide antibodies (ApepA) are specific antibodies that are used to target specific portions of the connexin proteins in the cell [77]. Antibodies can additionally inhibit hemichannels without affecting gap junction function because the antibodies cannot access all the proteins that form GJs [78].

Polyclonal antibodies have been used in rat hearts to study cell-to-cell communication. Antibodies were constructed to bind intracellular amino acid sequences 5–7, 314–322, and 363–382 of protein Cx43. The first two antibodies did not interfere with cell-to-cell communication, but the third was able to block coupling in 50% of the injected cells [79]. In a similar work, antibodies to amino acids 113–123, 241–260, 283–298, and 346–360 were studied for their effect on the phosphorylation of Cx43 [80]. Two different antibodies were made to target the last 23 amino acids (360–382) of Cx43, resulting in the inhibition of gap junction uncoupling, an increase in the channels’ open time, and a change in their selectivity [81]. In addition, ApepA were used against the extracellular loops of Cx proteins in human cells. This resulted in the total inhibition of the hemichannels, without affecting cell-to-cell coupling [82].

ApepA against Inx2 of *Drosophila* blocked the GJCs between oocytes and follicle cells in the intracellular C-terminus and the intracellular loop [83]. Previous works used antibodies to localize the expression of innexins in tissue [84], where they showed that the gap junction protein innexin-2 is expressed in a small group of nerve cells in the lower body column of invertebrates and that an anti-innexin-2 antibody binds to gap junctions in the same region. However, they did not use gene shutdown or innexin inhibition.

### 5.4. Antisense Oligonucleotides

Antisense oligonucleotides (ASOs) are short DNA sequences specifically designed to target the mRNA transcripts of specific proteins in order to decrease or abolish the expression of such proteins [85,86]. Direct applications of the use of ASOs in studying neurodegenerative diseases have been reviewed [85,86,87].

ASOs have been used with a Pluronic F-127 gel delivery system to regulate specific connexin expression. They are injected directly into tissues, resulting in connexin knockdown for 24–48 h [88]. The role of Cx43 was investigated using ASODs in a rodent model of optic nerve damage with and without modulation expression, resulting in a knockdown of Cx43 production and a decrease of new GJs, which reduced cell death and optic nerve oedema [89]. A similar study used a model of corneal wound healing to estimate the effect of Cx43 using ASOs. The knockdown of Cx43 resulted in faster wound closure and more uniform repair [90].

ASOs are effective when administered throughout the transcription of connexin genes and have no effect blocking connexin channels that already exist. In addition, ASOs need to be around 18–30 bases long. If they are longer, cell penetration is not possible. If they are shorter, they will be less specific. The advantages of ASOs are that they are easy to use, dose controllable, and low-cost compared to other gene knockout protocols [88].

### 5.5. Mimetic Peptides

Mimetic peptides (MPs) are synthetic peptides that assume a configuration compatible with a specific protein and inhibit channel formation by imitating connexin–connexin binding. Nonetheless, MPs are able to create channels on their own [91,92].

It was confirmed that MPs specific to the second extracellular region of connexins can inhibit specific types of gap junction channels [93]. MPs have been used to interrupt GJC in endothelial muscle and homocellular muscle culture systems. In addition, it was proven that peptides are reversible following a washout treatment [94].

Although MPs can inhibit gap junction channels, some evidence suggests a lack of inhibition of channel currents (less than 30%) [68]. Likewise, connexin MPs seem to inhibit pannexin channels, which are different in sequence from connexins [53]. Based on the results from those studies, MPs are not suitable for the straightforward inhibition of gap junction channels. MPs can only block the formation of new GJs but do not affect connections that are already formed.

## 6. Computational Models

Computational techniques have been useful in studying biological processes [95,96]. Recent evidence discusses the use of various computational approaches to simulate the molecular flux through connexin hemichannels using the structure of the pores obtained by X-ray crystallography and assuming Brownian dynamics for the molecules in flux [97,98,99,100]. In addition, an automated fluorescence microscope technique was developed to quantify gap junction communication using the values for nucleus number, cytoplasm area, cell perimeter, and fluorescence intensity from each cell in order to be able to recognize gap junction blockers [101]. Furthermore, a computational model was created to demonstrate the degradation of GJs inside the ischemic area in cardiac cells [102].

Machine learning is one of the fields of artificial intelligence that focuses on providing tools for data analysis. Those tools are often fast and scalable, but a large data set is expected to permit greater learning and prediction [103,104]. Machine learning has been used in engineering and in the natural sciences (physics, chemistry, and biology) and can help in the life sciences by providing useful computational models for neuroscience. The data used can diversify from small molecules to omic data (e.g., genomic, proteomic, transcriptomic, metabolomic) [105,106]. Nevertheless, further applications of machine learning to the study of gap junction channels is necessary due to the lack of research involving computational approaches in the study of innexin and connexin channels.

Support vector machines (SVMs) have been used to predict the different types of proteins as they have considerable accuracy for differentiating type I transmembrane, type II transmembrane, multipass transmembrane, lipid-chain anchored membrane, and GPI-anchored membrane [107]. An SVM model was generated to predict the secondary structure of proteins [108]. The same method was used to create a detector of membrane activity in α-helical peptide sequences [109] and to create a classifier to differentiate the redox states in molecular dynamics of proteins [110]. In addition, machine learning approaches regarding the study of proteins have been developed to predict DNA-protein binding sites [111], protein ligand biding affinity [112], the relationship between primary and secondary structure of globular proteins [113], protein sorting signals based on the sequence of amino acids [114], and many more.

There is a lack of machine learning applications regarding the inhibition or blockage of GJs. Nevertheless, in studies where confocal microscopy was used to study effects of ApepA in GJs from the myocardium [115], inverted microscopy was used to analyze infarct reduction by gap junction inhibition with octanol [116]. Fluorescence microscopy images were taken to study the effects of gap junction blockade in the suppression of central nervous system diseases [117]. There is benefit in the use of machine learning techniques, specifically convolutional neural networks, to process images. Convolutional neural networks have been successful in pattern classification and detection in natural images [104].

Related to metabolic applications, machine learning was used to develop a model to discriminate between related genotypes using metabolome analysis data [118]. An algorithm was developed to determine the metabolism and toxicity of new compounds [119]. The prediction of the biological function of compounds of metabolic pathways was achieved to predict what metabolic pathway a molecule belongs to [120]. A machine learning tool was trained to classify between essential and non-essential reactions using topologic, genomic, and transcriptomic features [121]. In addition, a machine learning method was used to predict metabolic pathways based on genome data [122] and to study drug metabolic process using gene cancer data [123].

Machine learning approaches are of particular interest in drug development due to their applicability in several steps of drug discovery methodology [124]. An SVM was used to predict the cleavability of oligopeptides to HIV proteases [125]. A machine learning approach was used to discern between substrates from an inhibitor of carrier proteins to be used in drug development [126]. Lastly, the SVM was trained with energy terms from docking sites to predict binding affinity and its application to drug biding affinity [127].

Cell-to-cell communication has a significant role in tumor differentiation and proliferation. Connexins containing GJs, tunneling nanotubes, and hemichannels are part of that type of communication. However, new approaches (such as machine learning) could provide new insights to the study of those signals between connected cells [128]. Nonetheless, there is little evidence of the use of machine learning techniques in cell-to-cell communication studies.

A machine learning strategy for studying Cx39 hemichannel permeability to certain molecules has been developed. Since the net charge, size, and shape are insufficient properties for determining pore affinity to certain molecules, the model consisted of 11 descriptors belonging to six categories: electronegativity, ionization potential, polarizability, size and geometry, topological flexibility, and valence [129].

## 7. Discussion and Future Work

Gap junction channels allow for the exchange of different metabolites and currents among cells. GJs are formed from proteins encoded by three types of genes: innexins in invertebrates and connexins and pannexins in vertebrates. Innexins and connexins have been identified throughout several animal models, where different species share similarities in the biological functions performed by the proteins encoded by those genes.

The medicinal leech is a suitable biological model for studying the nervous system, partly due to the similarities between human connexins and leech innexins [130]. Research has focused on the mechanisms of synaptic transmission and neurochemistry. In this regard, the leech seems to be an appropriate model for studying the behaviors of human connexins in a simpler living model.

Several studies have used rodents to study the functions of innexins or connexins using different methods aimed to inhibit the formation of GJs. Those methods vary in their specificity toward the entire protein or small sequences of amino acids. Other methods use different molecules to target protein mRNA and inhibit the translation of the targeted gene. Thus, the selection of a method to block GJs should be specific to the expected results.

The applications of machine learning to biological data and problems extends over several areas related to the study of GJs. There are different applications of machine learning techniques to study protein structure and interactions with other molecules. Research involving innexin and connexin proteins could be strengthened with the use of machine learning in order to improve structural and functional studies. In addition, the development of novel drugs that target gap junction genes or proteins could benefit from algorithms that predict the binding affinity of some molecules to other molecules and predict how a molecule will function in a specific metabolic pathway. Furthermore, given that several biological studies benefit from the use of microscopic images, these studies could use image processing algorithms, like convolutional neural networks, to detect patterns or classify images of biological tissue.

Even though in vivo and in vitro experimentation are the traditional ways to do research, computational approaches have been successful in different applications in biological fields, creating an important new arena for machine learning techniques. This gives scientists an opportunity to use different computational techniques to study gap junction related behavior. In silico experimentation is an advantageous approach for studying different biological processes as it reduces the time and resource consumption needed for in vitro experimentation.

Machine learning offers new opportunities in studying connexins and innexins, GJs, membrane flux of molecules, GJ blockers, etc., particularly to complement and expand what is already known in the field. Multidisciplinary research is the key to new developments involving applications of novel computational approaches to the understanding of current biological questions. Therefore, this research improves the quality and reach of these studies and scientific publications.

## Abbreviations

GJs	Gap junctions
CJC	Gap junction communication
RNAi	RNA interference
dsRNA	Double-stranded RNA
ApepA	Anti-peptide antibodies
ASOs	Antisense oligonucleotides
MPs	Mimetic peptides
SVM	Support vector machine
